# Dexmedetomidine Reduces Chronic Stress–Related Thrombosis in a Mouse FeCl_3_ Model

**DOI:** 10.1096/fj.202500724R

**Published:** 2025-04-30

**Authors:** Huazhen Wang, Meiping Zhang, Minglong Xin, Xueling Yue, Jinshun Piao, Longguo Zhao, Hehui Bi, Shiyan Wang, Chunzi Jin, Yongshan Nan, Xianglan Jin, Xian Wu Cheng

**Affiliations:** ^1^ Department of Anesthesiology Yanbian University Hospital Yanji Jilin P.R. China; ^2^ Department of Cardiology and Hypertension, Jilin Provincial Key Laboratory of Stress and Cardiovascular Disease Yanbian University Hospital Yanji Jilin P.R. China; ^3^ Key Laboratory of Natural Medicines of the Changbai Mountain, Ministry of Education Yanbian University Yanji Jilin P.R. China; ^4^ Department of Community Healthcare and Geriatrics Nagoya University Graduate School of Medicine Nagoya Japan

**Keywords:** apoptosis, chronic stress, dexmedetomidine, protease‐activated receptor‐2, thrombosis

## Abstract

Chronic psychological stress (CPS) is a significant risk factor for thrombotic cardio‐cerebrovascular diseases (TCVDs). Clinical data suggest that the α_2_‐adrenergic receptor (AdR‐α_2_) agonist dexmedetomidine (Dex) can influence coagulation in stress‐exposed intensive care unit patients. Given the important role of protease‐activated receptor‐2 (PAR‐2) in vascular pathobiology, we aimed to investigate the potential effects of Dex on stress‐related thrombus formation, focusing on the PAR‐2 signaling pathway. Eight‐week‐old male mice underwent non‐stress and immobilization stress with Dex treatment for 2 weeks and were then subjected to carotid artery thrombosis induction using ferric chloride (FeCl_3_). On Day 14 post‐stress, the mice exhibited increased thrombus weight and length, along with harmful alterations in the plasma levels of von Willebrand factor and metalloproteinase with thrombospondin Type 13 motifs. Additionally, arterial protein and/or mRNA levels of PAR‐2, p‐Akt, Bcl‐2, cleaved caspase‐3, cytochrome *c*, gp91^phox^, TNF‐α, MCP‐1, ICAM‐1, VCAM‐1, and TLR‐4 were altered, accompanied by arterial endothelial loss. Dex treatment reversed these changes. Conversely, AdR‐α2 blockage with yohimbine diminished the benefits of Dex. In vitro, Dex reduced stress serum‐induced reactive oxygen species production and endothelial apoptosis, along with beneficial alterations in PAR‐2, Bcl‐2, and cytochrome *c* protein levels. Yohimbine diminished these effects. Thus, α_2_‐adrenergic receptor activation appeared to mitigate stress‐related thrombus formation in mice undergoing FeCl_3_‐induced surgery, possibly by negatively regulating PAR‐2 signaling. These findings suggest a potential therapeutic strategy for CPS‐related thrombotic events in patients with TCVDs.

## Graphical abstract

Schematic summary of the mechanism by which dexmedetomidine prevents chronic stress–related carotid artery thrombosis in a mouse FeCl_3_ model. These findings present evidence and a potential mechanistic explanation of the Dex‐mediated amelioration of arterial thrombosis associated with the reduction of oxidative stress, inflammation, and apoptosis, and proteolysis, that was mediated by PAR2 signaling inactivation in mice exposed to our experimental stress conditions. ADAMTS13, A disintegrin and metalloproteinase with thrombospondin Type 1 Motif 13; Dex, dexmedetomidine; PAI‐1, plasminogen activator inhibitor‐1; p‐Akt, phosphorylated protein kinase B; PAR2, protease‐activated receptor‐2; vWF, von Willebrand factor; Yoh, yohimbine.

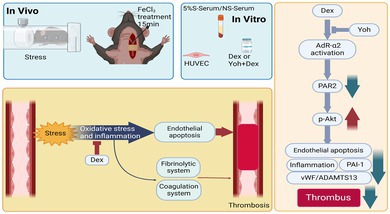

**Abbreviations**
ADAMTS13: a disintegrin and metalloproteinase with thrombospondin type 1 motif 13
AdR‐α2: α2‐adrenergic receptor
Bcl‐2: B‐cell lymphoma‐2
CPS: chronic psychological stress
EBM‐2: endothelial basal medium‐2
EGM‐2: endothelial growth medium‐2
GAPDH: glyceraldehyde 3‐phosphate dehydrogenase
H&E: hematoxylin–eosin
ICAM‐1: intercellular adhesion molecule‐1
MCP‐1: monocyte chemoattractant protein‐1
NF‐κB: nuclear factor kappa B
NLRP3: NOD‐like receptor protein 3
p‐Akt: phosphorylated protein kinase B
PAR‐2: protease‐activated receptor‐2
PI3K: phosphatidylinositol 3‐kinase
qPCR: quantitative polymerase chain reaction
ROS: reactive oxygen species
TCVDs: thrombotic cardio‐cerebrovascular diseases
TLR‐4: toll‐like receptor‐4
TNF‐α: tumor necrosis factor‐α
TUNEL: terminal deoxynucleotidyl transferase dUTP nick‐end labeling
VCAM‐1: vascular cell adhesion molecule‐1
vWF: von Willebrand factor

## 1 Introduction

Chronic psychological stress (CPS) is closely associated with the incidence of many diseases, including neurodegenerative disorders, cancer, diabetes mellitus, and thrombotic cardio‐cerebrovascular diseases (TCVDs) [1, 2, 3, 4]. CPS is a significant and often underappreciated risk factor for thrombosis in both healthy individuals and patients with preexisting cardiovascular conditions [5]. Emerging evidence indicates that CPS exacerbates prothrombotic states by promoting endothelial dysfunction, platelet hyperactivity, and inflammatory responses [6, 7]. Furthermore, studies have demonstrated that prolonged stress exposure increases the risk of arterial and venous thrombus formation, particularly in animal models and human studies of TCVDs [8]. These findings underscore the importance of elucidating the precise mechanisms underlying CPS‐induced thrombosis to inform potential therapeutic strategies.

Dexmedetomidine (Dex) is a highly selective α2‐adrenergic receptor (AdR‐α_2_) agonist widely used for sedation and analgesia, especially in the intensive care unit (ICU) setting [9]. As a member of the AdR family, AdR‐α_2_ is extensively expressed in immune tissues, where it plays key roles in modulating inflammatory and immune responses [10]. Recent literature has emphasized Dex's potent anti‐inflammatory properties, with accumulating evidence supporting its therapeutic potential in various inflammatory and cardiovascular injuries [11]. Experimental findings suggest that moxonidine enhances the uptake of oxidized low‐density lipoprotein by vascular smooth muscle cells through AdR‐α2 activation, thereby regulating inflammation and attenuating atherosclerotic plaque growth induced by Angiotensin II infusion [12]. Interestingly, clinical data analysis also reveals that Dex reduces hypercoagulability in ICU patients undergoing prolonged sedation [9]. Further exploration is needed to elucidate the precise mechanisms by which it exerts these vascular protective effects.

Protease‐activated receptors (PARs), belonging to the G protein–coupled receptor family, are characterized by their seven transmembrane domains. They are activated by proteolytic cleavage of their N‐terminal regions by specific proteases, generating new tethered ligands that activate the receptor intramolecularly [13]. Among the four identified PAR subtypes, PAR‐2 has emerged as a principal receptor for activated factor X (FXa), being prominently expressed in vascular endothelial cells, smooth muscle cells, and leukocytes, but notably absent in platelets [14]. Evidence indicates that PAR‐2 plays a critical role in regulating inflammation, contributing to the pathophysiology of various inflammatory conditions within endothelial cells [15, 16]. Notably, PAR‐2 has been shown to induce reactive oxygen species (ROS)‐mediated inflammation through Akt‐driven modulation of the nuclear factor kappa B (NF‐κB) and FoxO6 signaling pathways, highlighting its role in oxidative stress and inflammatory cascades [17]. Despite the well‐established interplay between inflammation and coagulation, the precise role of PAR‐2 in the development and progression of thrombotic diseases remains largely elusive, warranting further investigation.

Given that the ferric chloride (FeCl_3_)‐induced thrombosis model is frequently used to study arterial thrombus formation and is often applied to screen drugs and explore their molecular mechanisms, we used chronic stress along with a murine thrombosis model of FeCl_3_ induction to examine Dex‐mediated prevention of chronic stress–induced carotid artery endothelial damage and thrombus formation, in combination with the AdR‐α2 antagonist yohimbine, in vivo and in vitro.

## 2 Materials and Methods

### 2.1 Materials

Commercial antibodies were utilized in this study. The following antibodies were purchased from Cell Signaling Technology (Danvers, MA, USA): anti‐Akt (protein kinase B) antibody (Cell Signaling Technology, catalog #2967), anti‐phosphorylated Akt (p‐Akt (S473), #4060), anti‐PI3K (phosphatidylinositol 3‐Kinase, catalog #4292), anti‐phosphorylated PI3K (p‐PI3K p85 (Tyr458)/p55 (Tyr199) #17366), anti‐cleaved caspase‐3 (c‐casp3, #9664), anti‐cytochrome c (#11940), anti‐B‐cell lymphoma 2 (Bcl‐2, #2870), anti‐Plasminogen Activator Inhibitor‐1 (PAI‐1, #27535), anti‐glyceraldehyde 3‐phosphate dehydrogenase (GAPDH, #5174), anti‐mouse IgG (#14709), anti‐rabbit IgG (#14708), anti‐rat IgG (#4416), and anti‐mouse IgG (#5470). The immunohistochemical staining kit (PV‐9000) was purchased from ZSGB‐BIO (Beijing, China). The following antibodies were purchased from BD Pharmingen (San Diego, CA, USA): anti‐gp91^phox^ (#611415), anti‐CD31 (#550274), and anti‐SOD2/MnSOD (#ab13534). The ABC Kit Peroxidase (#PK‐4004) and DAPI (H‐1200) were purchased from Vector Laboratories (Burlingame, CA, USA). The enzyme‐linked immunosorbent assay (ELISA) kits for a disintegrin‐like and metalloproteinase with thrombospondin type 1 motifs (ADAMTS13, JL43139) and von Willebrand factor (vWF, JL20631) were purchased from JONLNBIO (Shanghai, China). The RNeasy Micro Kit and SYBR Green Master Mix were obtained from Qiagen (Hilden, Germany). The SuperScript III First Strand was purchased from Invitrogen (Carlsbad, CA, USA). The RIPA lysis buffer (R0010) and BCA protein assay kit were purchased from Solarbio (Beijing, China). The polyvinylidene fluoride (PVDF) membrane was from Merck Millipore (Carrigtwohill, Ireland), and the Amersham ECL Prime Western Blotting Detection kit was sourced from GE Healthcare (Freiburg, Germany). The OCT compound was purchased from Sakura (Finetechnical, Tokyo, Japan). Endothelial basal medium‐2 (EBM) and endothelial growth medium‐2 SingleQuots (EGM) were purchased from Lonza (Walkersville, MD, USA). The non‐fluorescent probe 2′,7′‐dichlorofluorescein diacetate (DCFH‐DA) was obtained from Beyotime (S0033M, Shanghai, China). The In Situ Cell Death Detection Kit (#11684795910) was purchased from Roche Diagnostics (Mannheim, Germany).

### 2.2 Network Pharmacology Analysis

#### 2.2.1 Screening of Dexmedetomidine Component Targets and Thrombosis‐Related Targets

The chemical structure of Dex was imported into the Swiss Target Prediction and SuperPred databases to search for potential targets. After merging and removing duplicates, the resulting list identified potential targets of Dex. Additionally, the keyword “thrombosis” was employed to search for disease‐related targets in the DisGeNET and GeneCards databases. The retrieved targets were merged, duplicates were eliminated, and thrombosis‐related targets were further refined through a literature review.

#### 2.2.2 Construction and Analysis of Protein–Protein Interaction (PPI) Network

The intersection of Dex component targets and thrombosis‐related targets was identified. These intersecting targets were imported into the STRING database, with the species set to *Homo sapiens* and the minimum required interaction score set to medium confidence (0.400). The resulting TSV file was imported into Cytoscape 3.10.2 for visualization and analysis. The “Network Analyzer” plugin was utilized to calculate topological parameters, and targets with a degree value (Degree) greater than or equal to the average were designated as core targets for Dex in the treatment of thrombosis.

#### 2.2.3 KEGG Pathway Enrichment Analysis

The key targets derived from the intersection of drug targets and disease targets were imported into the Metascape database for KEGG pathway enrichment analysis. The enriched pathways were visualized using the Omicshare platform (https://www.omicshare.com/), with the species limited to *Homo sapiens* and a significance threshold of *p* < 0.05. The enriched genes were ranked by enrichment score to obtain biological information and functional annotations of the key targets, enabling the analysis of the potential mechanisms of Dex in treating thrombosis.

### 2.3 Retrospective Analysis of the MIMIC Clinical Database

A retrospective study was conducted utilizing data from the Medical Information Mart for Intensive Care (MIMIC‐IV) database (see: https://mimic.mit.edu). Patients with a hospital stay of at least 7 days were included, and coagulation‐related parameters, including prothrombin time (PT), partial thromboplastin time (PTT), and international normalized ratio (INR), were extracted for analysis.

### 2.4 Animal Care and Use

Male wild‐type C57BL/6J mice, aged 8 weeks and weighing 20–23 g, were housed under standard conditions (23°C ± 1°C, 50% ± 5% humidity, 12‐h light/dark cycle) in individually ventilated cages. Mice were provided with standard chow and tap water. Animal experiments were conducted in accordance with the Institutional Animal Care and Use Committee of Yanbian University [18]. Mice were closely monitored during the study, and all procedures involving them were carried out by qualified researchers. All experimental protocols were approved by the Institutional Animal Care and Use Committee of Yanbian University (protocol no. YD20211128002).

### 2.5 Chronic Restraint Stress Protocol and Induction of Carotid Artery Thrombosis

Eight‐week‐old mice were randomly assigned to five groups: control (Cont), control + Dex (C‐Dex), stress + NaCl (Stress), stress + Dex (S‐Dex), and stress + yohimbine + Dex (S‐Dex + Yoh). NaCl, Dex, or yohimbine was administered intraperitoneally every morning before stress exposure. Mice underwent a restraint stress program as described previously [19]. Between 8:00 AM and 12:00 PM, the stress group mice were individually placed in appropriately sized restrainers to limit movement for 4 h per day, with no access to food or water. Control mice were allowed to move and interact freely without disturbance. This stress protocol was followed for 2 weeks.

On Day 14 of chronic restraint stress, the FeCl_3_‐induced thrombosis model of the mouse carotid artery was established as described [20]. In brief, each mouse was anesthetized with 5% isoflurane inhalation via an air delivery system. After the mouse became unconscious, the anesthetic agent was switched to 2%–3% isoflurane to maintain anesthesia [21]. After exposure of the left carotid artery, a 1 × 2 mm filter paper soaked in 20% FeCl_3_ was applied to the artery for 15 min, followed by careful dissection of the vessel. When the vessel wall changed to a darker color, both ends of the filter paper strip were ligated to accurately collect the artery.

### 2.6 Sample Collection

Mice underwent a 14‐day stress protocol with or without pharmacological intervention. On the final day, thrombus formation was induced in the left carotid artery using filter paper saturated with 20% FeCl_3_ solution as described above. The artery was then ligated at both ends and excised, and its length and weight were measured. Blood samples were obtained from the left ventricle of each mouse and analyzed using ELISA. Arteries were preserved in RNAlater for subsequent gene expression analysis or promptly snap‐frozen in liquid nitrogen for protein assessment. For histopathological evaluation, arteries were promptly embedded in optimal cutting temperature (OCT) compound, frozen in liquid nitrogen, and sectioned using a cryostat. The prepared arterial sections were used for hematoxylin–eosin (H&E) staining and immunohistochemistry.

### 2.7 Gene Expression Analysis

Total RNA was isolated from carotid artery tissue using the RNAeasy Mini Kit, following the manufacturer's instructions [22]. Complementary cDNA was synthesized with SuperScript III reverse transcriptase. Quantitative PCR was conducted using Power SYBR Green PCR Master Mix on the ABI 7300 real‐time PCR system (Applied Biosystems, Foster City, CA, USA). Gene expression was quantified using the 2^−ΔΔCt^ method, with all experiments performed in triplicate. mRNA levels were normalized to GAPDH.

### 2.8 Western Blot Analysis

Fresh carotid artery tissue or cells were lysed in RIPA buffer containing 1% PMSF and 1% protein phosphatase inhibitor mixture (P1260, Solarbio) to extract total protein. Protein concentrations were determined using the BCA Protein Assay Kit by measuring absorbance at 562 nm with a microplate reader [23]. Equivalent protein amounts (30 μg per lane) were resolved by SDS‐PAGE, transferred onto PVDF membranes, and blocked with 5% non‐fat milk prepared in TBS‐Tween. Membranes were incubated overnight at 4°C with primary antibodies (1:1000), followed by a 2‐h incubation at room temperature with secondary antibodies (1:15 000). Target proteins were visualized using the Amersham ECL Prime Western Blotting Detection Reagent, with GAPDH serving as the loading control.

### 2.9 Morphological Assessment and Immunohistochemical Analysis

Vascular tissues were sectioned into 45‐μm‐thick cryosections for histological evaluation, followed by H&E staining. To assess endothelial injury or loss, immunohistochemical staining for CD31^+^ was performed on arterial sections from stress and thrombosis induction at Day 21. After washing twice with phosphate‐buffered saline (PBS), tissue sections were incubated with mouse IgG (1:200) for 1 h, and CD31^+^ expression was visualized using an ABC substrate kit (Vector Laboratories). ImagePro software (BZ‐9000 Analysis, Keyence, Osaka, Japan) was used to analyze the CD31^+^ immunostained images. Cross‐sectional areas of each vessel (*n* = 5–7) were examined, and the average value per mouse was calculated. Data are presented as the number of CD31^+^ cells within the endothelial region (0.5 mm^2^) of the lesions [24].

### 2.10 Immunofluorescence Assay

Carotid artery sections were blocked with 5% bovine serum albumin for 30 min, followed by overnight incubation with rabbit anti‐CD31^+^ polyclonal antibody (1:100). Sections were washed three times with PBS, incubated with an anti‐rabbit IgG fluorescent secondary antibody for 1 h at room temperature, and counterstained with DAPI in an anti‐fluorescence quenching solution. Images were acquired using the EVOS FL Auto 2 Imaging System (Thermo Fisher Scientific, Waltham, MA, USA) [25].

### 2.11 ELISA

Blood samples collected from the left ventricle of mice were used for ELISA. Plasma levels of vWF and ADAMTS13 were quantified using commercial ELISA kits according to the manufacturer's protocols [24].

### 2.12 Cell Culture

Human umbilical vein endothelial cells (HUVECs) were obtained from Cell Applications (San Diego, CA, USA) and used at passages 2–8. The cells were cultured in Endothelial Growth Medium‐2 (EGM‐2, Lonza) at 37°C in a humidified environment with 5% CO_2_ and 95% air. HUVECs were seeded at a density of 4 × 10^5^ cells per well in 6‐well plates and incubated for 24 h before proceeding with cell assays.

### 2.13 Assay of Intracellular Reactive Oxygen Species Production

Reactive oxygen species (ROS) production was detected using the non‐fluorescent probe 2′,7′‐dichlorofluorescein diacetate (DCFH‐DA). DCFH‐DA passively diffuses into cells and is deacetylated by intracellular esterases to form the non‐fluorescent compound 2′,7′‐dichlorofluorescein (DCFH). In the presence of ROS, DCFH reacts to produce the fluorescent product dichlorofluorescein (DCF), which is retained within the cells. After the corresponding treatments according to the groups, the culture medium was removed, and the cells were washed three times with PBS. DCFH‐DA was diluted to a final concentration of 10 μM with EBM, added to the culture medium, and incubated for 30 min at 37°C. Fluorescence intensity was measured using a fluorescence microscope with an excitation wavelength of 485 nm and an emission wavelength of 530 nm. The increase in fluorescence compared to the NS‐serum group was considered an indication of increased intracellular ROS.

### 2.14 Apoptosis Assay

Approximately 5–10 × 10^4^ HUVECs were seeded on 6‐well glass slides (Biogix, Jinan, China) and allowed to adhere overnight. After a 2‐h pre‐treatment with or without Dex or in combination with yohimbine, cells were cultured in endothelial basal medium‐2 (EBM‐2) containing 5% stress or non‐stress serum for 24 h before undergoing apoptosis assays. Apoptotic cells were quantified using an in situ cell death detection kit, following the manufacturer's protocol [26]. Cells were assessed in three randomly selected regions on the slides, and apoptotic cells were quantified using terminal deoxynucleotidyl transferase dUTP biotin nick‐end labeling (TUNEL).

### 2.15 Statistical Analysis

Results are presented as mean ± SEM, with sample sizes (*n*) provided in the figure legends and experimental descriptions. Data normality was assessed using the Shapiro–Wilk test. For non‐normally distributed datasets, the Kruskal–Wallis test was used. For normally distributed datasets, statistical analyses included a two‐tailed unpaired Student's *t*‐test and one‐way ANOVA, followed by Tukey's post hoc test for multiple comparisons. For grouped datasets, two‐way ANOVA was performed, followed by Tukey's multiple comparisons test. For groups with fewer than five samples, pairwise comparisons were conducted using the Mann–Whitney *U*‐test and the rank‐sum test. For analyses involving multiple groups, the Kruskal–Wallis test combined with Tukey's post hoc analysis was applied. Unless otherwise specified, each experiment was independently replicated at least three times. Mice were randomly allocated to experimental groups. Morphological analyses were conducted independently by two blinded observers, and their values were averaged. Sample size calculations were not performed using statistical methods, and no data points were excluded from the analysis. Statistical evaluations were conducted using GraphPad Prism version 9.0.0, with a significance threshold of *p* < 0.05.

## 3 Results

### 3.1 MIMIC‐IV Database Analysis Shows Dexmedetomidine Ameliorates Hypercoagulability in ICU Patients

We extracted coagulation‐related data (PT, PTT, INR) from the MIMIC‐IV database for patients with a hospital stay longer than 7 days [27]. Data were grouped based on the use of Dex (Table 1). The results indicated that long‐term ICU hospitalization led to a hypercoagulable state, and a comparison of the final coagulation measurements showed that Dex treatment reduced hypercoagulable changes in the blood (Table 1).

**TABLE 1:** Coagulation parameters.

Check the metrics	Initial examinations (*n* = 7231)	Final examinations (*n* = 7231)	*p*	Control group (*n* = 4183)	Dex group (*n* = 3048)	*p*
PT	13.9 (9.6, 18.2) s	13.6 (9.8, 17.4) s	< 0.001	13.5 (9.7, 17.3) s	13.6 (9.6, 17.6) s	0.021
PTT	30.7 (19.6, 41.8) s	31 (21.1, 40.9) s	< 0.001	30.9 (20.8, 41) s	31.1 (21.6, 40.6) s	0.102
INR	1.49 ± 0.87	1.45 ± 0.78	< 0.001	1.45 ± 0.85	1.46 ± 0.78	< 0.001

### 3.2 Network Pharmacology Analysis to Identify Targeted Molecules

**FIGURE 1:**
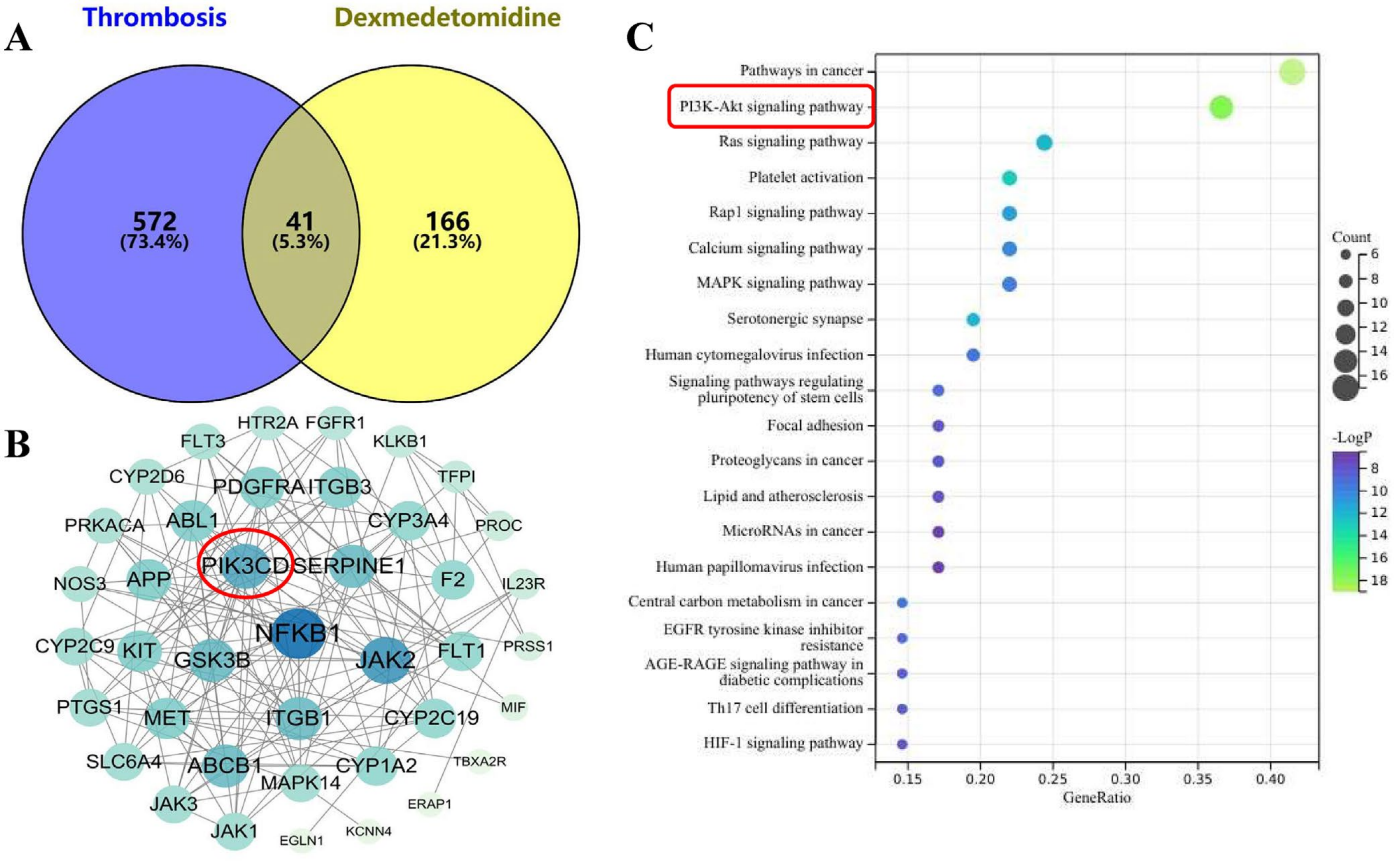
Analysis of targets associated with dexmedetomidine (Dex) and thrombosis. (A) Venn diagram illustrating the targets related to Dex and thrombosis. It includes 166 Dex‐related targets (right), 572 thrombosis‐related targets (left), and 42 targets associated with both Dex and thrombosis (center). (B) OA anti‐thrombosis PPI network. A larger area indicates larger nodes; blue color indicates a higher association, while lighter colors indicate less association. The core target is represented in the inner circle. (C) KEGG pathway enrichment analysis (DAVID). The Y‐axis represents pathways, the X‐axis represents FDR, and *p* values are indicated by color changes. Bubble size reflects the number of genes enriched in each pathway.

To investigate the potential molecular targets of Dex in anti‐thrombosis, a network pharmacology analysis was conducted. A total of 166 genes associated with Dex were identified using the SwissTargetPrediction database, while 572 genes related to thrombosis were retrieved from the GeneCards database. Subsequently, a Venn diagram was constructed using the Bioinformatics platform, revealing 41 overlapping targets (Figure 1A). To visually evaluate the effects of Dex on thrombosis, these 41 common targets were imported into the STRING database, and a protein–protein interaction (PPI) network was constructed using Cytoscape 3.10.2 software. The size and color of the nodes were adjusted according to their degree values, with darker node colors indicating higher degree values (Figure 1B). The results showed that NFkB, JAK2, and PIK3CD had higher degree values.

To systematically explore the potential mechanisms of Dex in anti‐thrombosis, we performed KEGG pathway analysis to identify the signaling pathways associated with Dex's anti‐thrombotic targets. The analysis revealed 172 statistically significant pathways. Subsequently, we performed KEGG enrichment analysis on these anti‐thrombotic targets using the DAVID database. Of the 16 relevant pathways identified (Figure 1C), the top 3 were cancer pathways, the PI3K/Akt signaling pathway, and the Ras signaling pathway. These findings suggest that Dex may exert its anti‐thrombotic effects primarily through the PI3K/Akt and Ras signaling pathways, providing a theoretical foundation for further mechanistic exploration and therapeutic applications.

### 3.3 Dex Treatment Prevents Chronic Stress–Related Arterial Endothelial Injury and Thrombosis

**FIGURE 2:**
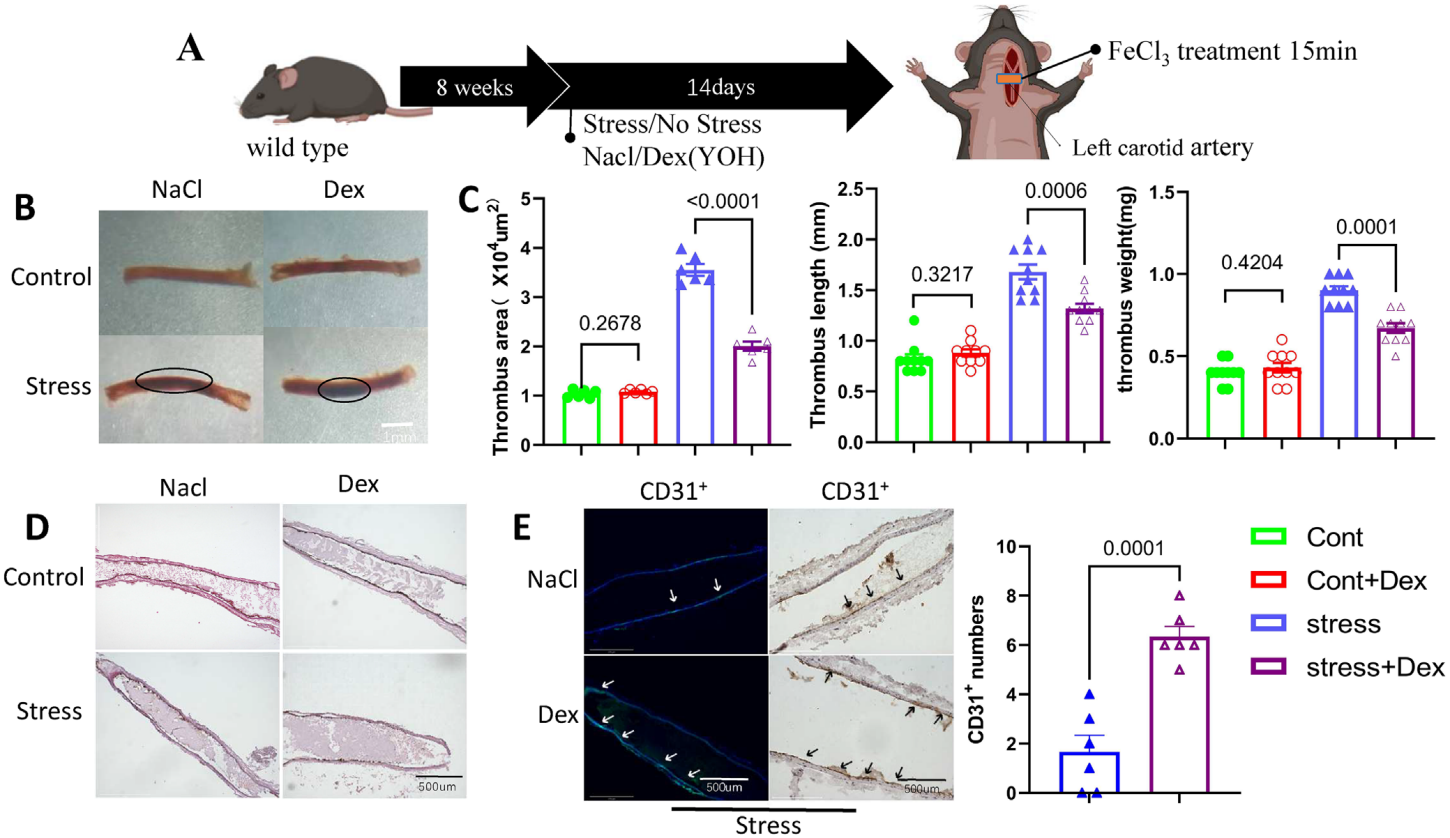
Dexmedetomidine alleviates FeCl_3_‐induced thrombus formation in stressed mice. (A) Schematic representation of the chronic stress model protocol and the arterial thrombosis induction process. (B and C) Representative photos and quantitative analysis of thrombus area, weight, and length across four experimental groups (*n* = 10/group for weight and length; *n* = 6/group for area). (D) Representative hematoxylin and eosin (H&E) staining images of thrombus, showing corresponding histological features. (E) Representative images (immunofluorescence: Left panels; immunostaining: Right panels) and quantitative data showing the number of CD31^+^ endothelial cells in the thrombi of two experimental groups (*n* = 6/group). Data are expressed as mean ± SEM, with statistical differences analyzed using one‐way ANOVA and Tukey's post hoc test for panel C, and unpaired Student's *t*‐tests for panel D. Scale bars: 1 mm and 500 μm.

To assess the impact of Dex on chronic stress–related endothelial injury and thrombosis, we applied a FeCl_3_‐induced thrombosis model to the carotid artery in mice subjected to restraint stress for 2 weeks (Figure 2A). The results indicated that stress significantly increased both thrombus weight and size while reducing mouse body weight. Dex treatment decreased thrombus weight and size without a significant change in body weight (Figure 2B,C). Quantitative analysis of H&E staining confirmed these findings (Figure 1D). As shown in Figure 1E,F, immunofluorescence and immunohistochemical staining of frozen sections of the carotid artery revealed a significant increase in the number of CD31^+^ endothelial cells following Dex treatment, indicating that Dex prevented the stress‐related arterial endothelial cell injury and thrombus formation induced by FeCl_3_.

### 3.4 Dex Reduces Inflammation, Oxidative Stress, Proteolysis, and Apoptosis In Vivo

**FIGURE 3:**
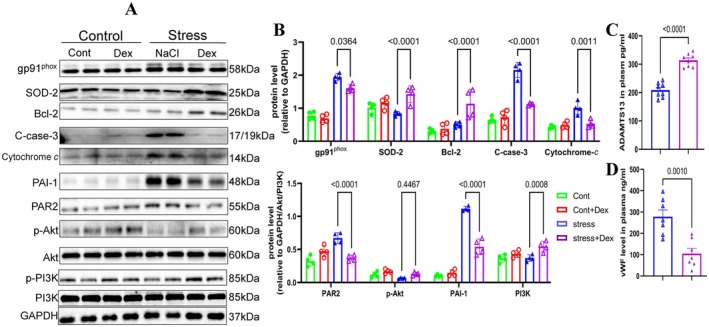
Dexmedetomidine reduces pathological protein expression and plasma hypercoagulability induced by chronic stress. Mice were intraperitoneally injected with NaCl or Dex and subjected to 4 h of daily chronic stress for 14 consecutive days. Carotid arteries were collected for protein analysis. Western blot analysis was performed on total proteins extracted from thrombosed carotid arteries. (A and B) Representative Western blots and quantitative data show the expression levels of gp91^phox^, SOD‐2, Bcl‐2, PAR‐2, PAI‐1, C‐cas‐3, cytochrome *c*, and p‐Akt across four experimental groups (*n* = 4/group). (C and D) Plasma concentrations of ADAMTS13 and vWF were assessed using ELISA after 14 days of chronic stress in four groups (*n* = 8/group). Data are expressed as mean ± SEM, with statistical significance determined by one‐way ANOVA and Tukey's post hoc test for panel B, and unpaired Student's *t*‐tests for panel C.

**FIGURE 4:**
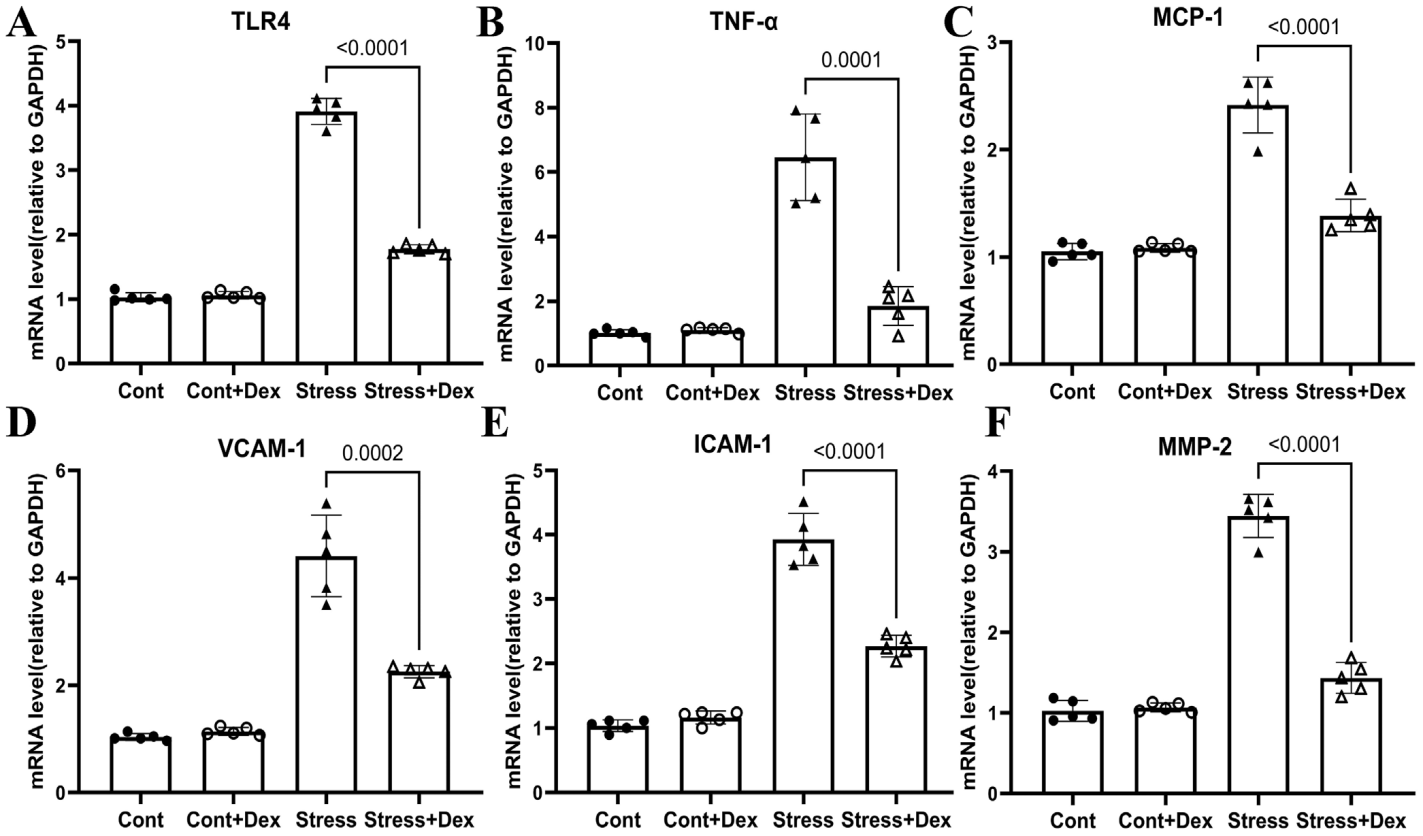
Dexmedetomidine reduces the expression of inflammation‐ and proteolysis‐related target genes in the carotid arteries of stressed mice. (A–F) QPCR analysis shows the levels of MMP‐2, TLR4, MCP‐1, VCAM‐1, ICAM‐1, and TNF‐α in four experimental groups (*n* = 5). Data are presented as mean ± SEM, and statistical significance was assessed using one‐way ANOVA and Tukey's post hoc test for panels A–F.

To investigate the mechanisms underlying Dex‐mediated protection against chronic stress–related thrombosis, we examined changes in proteins related to inflammation, oxidative stress, proteolysis, and apoptosis in non‐injured and injured arterial tissues of mice subjected to stress for 2 weeks. As anticipated, administration of Dex resulted in a reduction in the levels of gp91^phox^, cleaved caspase‐3, cytochrome *c*, PAI‐1, and PAR‐2 proteins, along with an increase in the levels of SOD‐2, Bcl‐2, p‐PI3K, and p‐Akt in the injured arteries of stressed mice (Figure 3A,B). We also observed a similar reduction in plasma levels of ADAMTS13 and vWF in thrombosed and stressed mice. qPCR analysis demonstrated that the levels of all investigated inflammation‐related mRNAs (VCAM‐1, MCP‐1, ICAM‐1, TLR4, and TNF‐α) and proteolysis‐related mRNA (MMP‐2) were lower in the Stress+Dex group compared to the stressed group alone (Figure 4). Thus, these results indicate that the anti‐oxidative stress, anti‐inflammatory, anti‐proteolytic, and anti‐apoptotic actions of Dex may contribute to vasculoprotection.

### 3.5 α2‐Adrenergic Receptor Antagonist Reverses the Protective Effects of Dex

**FIGURE 5:**
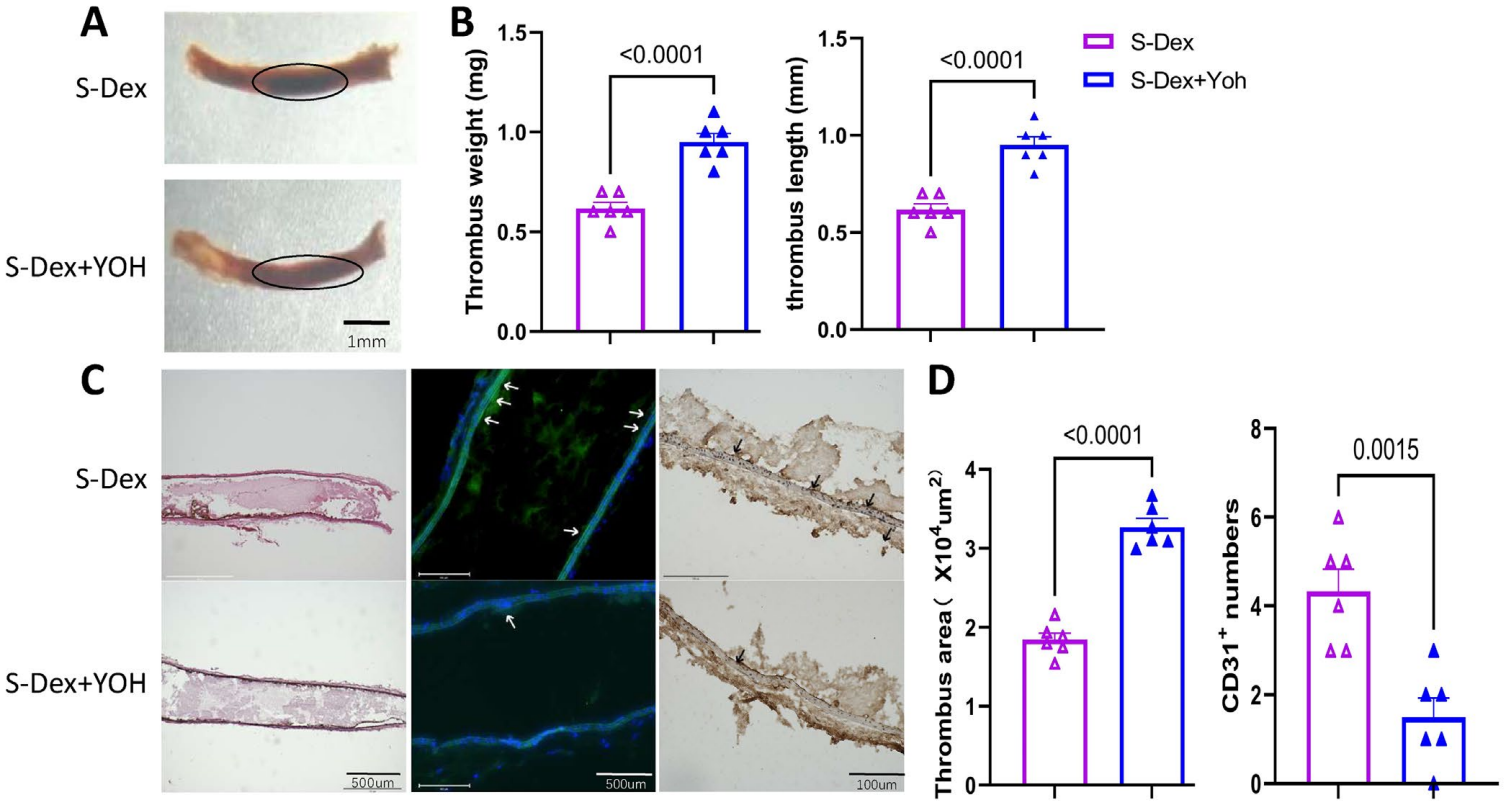
The α2‐adrenergic receptor antagonist yohimbine abrogates the protective effect of Dex on chronic stress‐induced endothelial damage and thrombus formation. Yohimbine was administered alongside Dex. After 14 days of stress, thrombosis was induced in the carotid artery using FeCl_3_ for 15 min, followed by experimental analysis. (A and B) Representative photos and quantitative data for the levels of wet thrombus length and weight in two experimental groups (*n* = 6). (C and D) Representative images (H&E staining, CD31^+^ immunofluorescence, and immunohistochemistry) and quantification of thrombus area and endothelial cell counts in the two experimental groups (*n* = 6). Data are presented as mean ± SEM and analyzed using unpaired Student's *t*‐tests. Scale bars: 1 mm, 500 μm, 100 μm.

**FIGURE 6:**
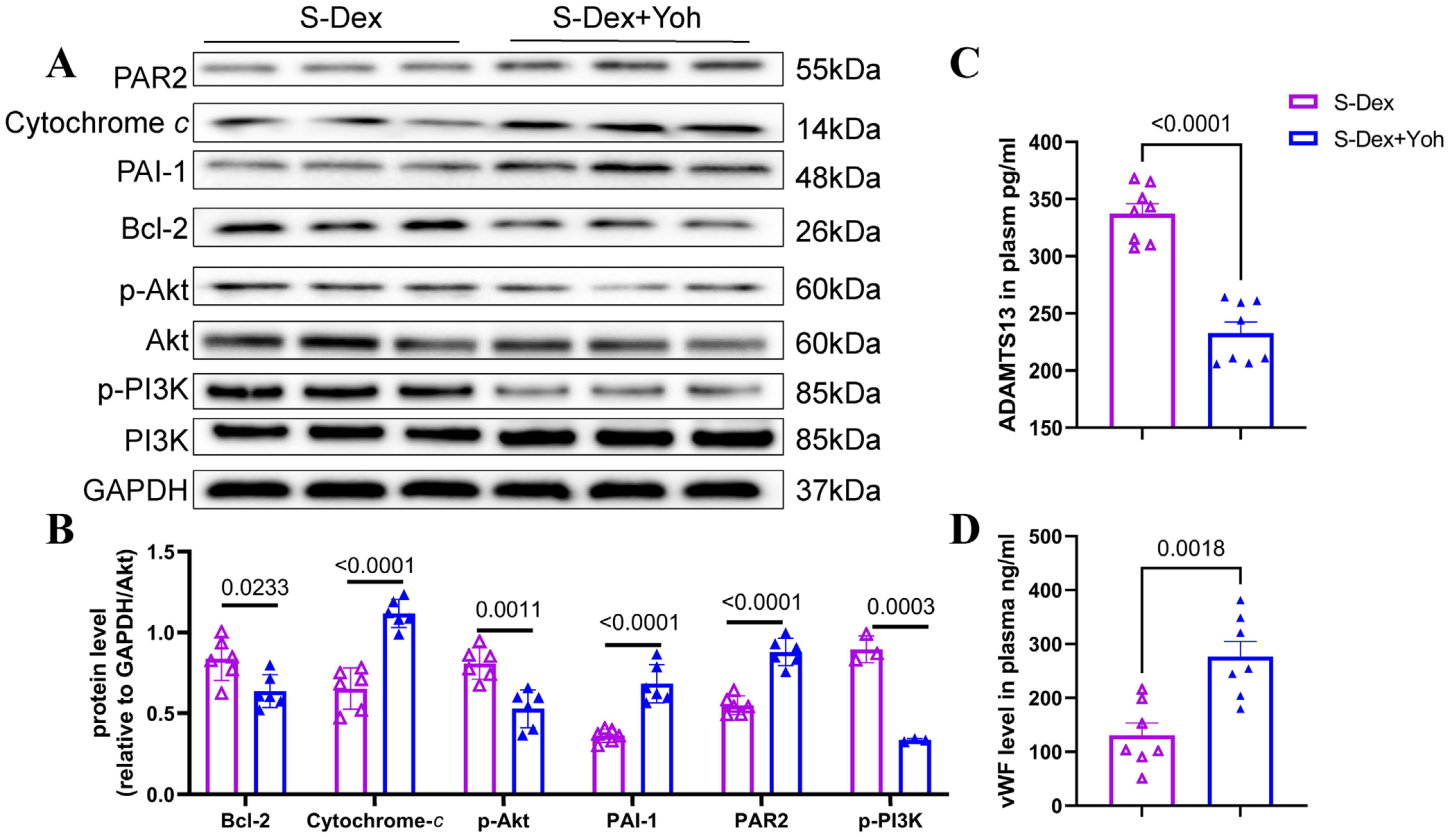
Yohimbine abrogates Dex‐mediated beneficial alterations in targeted protein levels in FeCl_3_‐treated arteries after 14 days of the stress protocol. (A and B) Representative images and quantitative data show the levels of Bcl‐2, cytochrome c, PAI‐1, PAR‐2, and P‐Akt in the thrombotic arterial tissues of two experimental groups (*n* = 6). (C and D) Plasma levels of ADAMTS13 and vWF were measured using ELISA in two groups (*n* = 8). Data are expressed as mean ± SEM and analyzed using unpaired Student's *t*‐tests for panels B and C.

**FIGURE 7:**
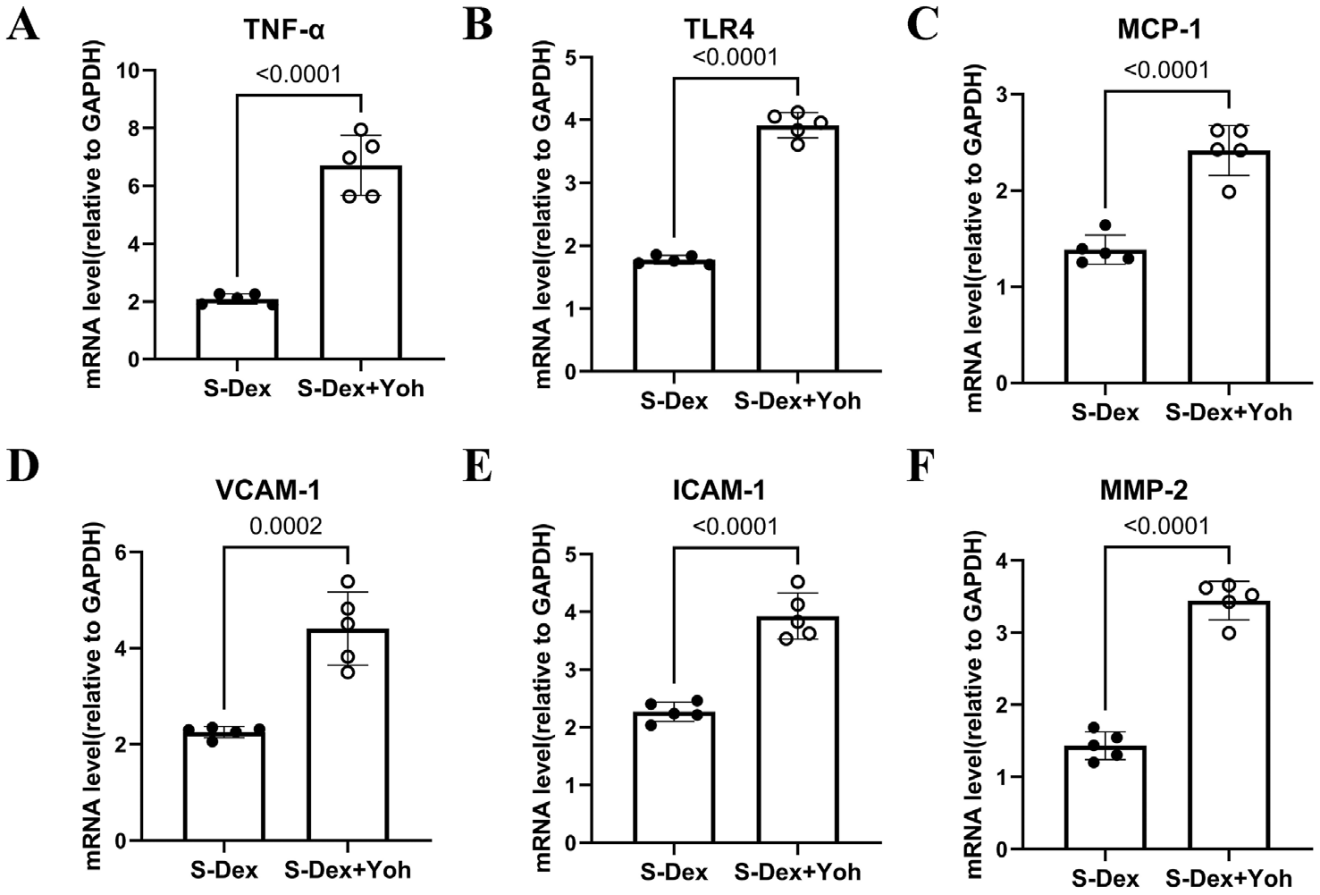
Yohimbine increases the expression of inflammation‐ and proteolysis‐related target genes in the carotid arteries of stressed mice treated with Dex. (A–F) QPCR analysis shows the levels of MMP‐2, TLR4, MCP‐1, VCAM‐1, ICAM‐1, and TNF‐α in four experimental groups (*n* = 5). Data are presented as mean ± SEM, and statistical significance was assessed using one‐way ANOVA and Tukey's post hoc test for panels A–F.

To investigate the role of α2‐adrenergic receptors in this process, we administered the specific α2‐adrenergic receptor antagonist yohimbine in conjunction with Dex. As shown in Figure 5A,B, α2‐adrenergic receptor blockade led to significantly increased thrombus weight and size, as well as an enlarged thrombus formation area compared to the Dex‐alone group. Additionally, yohimbine treatment reduced the endothelial cell count in the injured carotid artery (Figure 5C,D). As illustrated in Figure 6A,B, α2‐adrenergic receptor blockade increased the protein levels of PAR‐2 and the pro‐apoptotic cytochrome *c*, while reducing the protein levels of p‐PI3K, p‐Akt, and the anti‐apoptotic Bcl‐2 (Figure 6A,B). This blockade also resulted in harmful changes in plasma vWF and ADAMTS13 levels (Figure 6C,D). In parallel with these results, there was a significant increase in the levels of proteolysis‐related (MMP‐2) and inflammation‐related (TLR4, MCP‐1, VCAM‐1, ICAM‐1, and TNF‐α) genes (Figure 7).

### 3.6 Dex Alleviates Stress Serum–Induced Apoptosis in HUVECs by Activating α2‐Adrenergic Receptors

**FIGURE 8:**
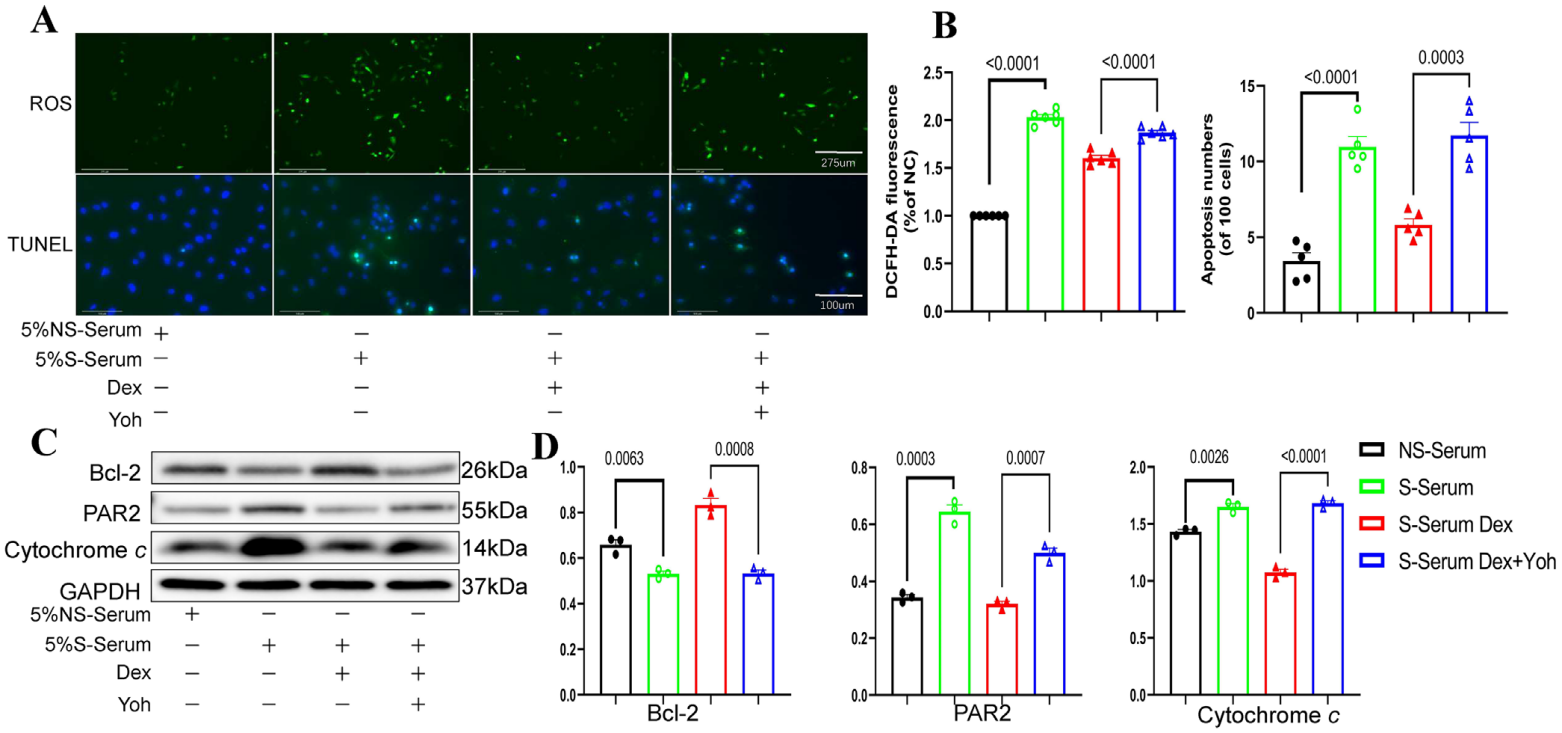
Dex treatment reduces stress serum–induced ROS production and apoptosis; these effects were diminished by yohimbine treatment. Following pretreatment with Dex, with or without yohimbine, for 30 min, the HUVECs were cultured in EBM‐2 containing 5% non‐stress serum (NS‐serum) and 5% stress serum (S‐serum), respectively, for 24 h. The cells were then subjected to evaluations of ROS production (immunofluorescence) and apoptosis (TUNEL) assays (*n* = 4 for each of the four groups). (A, B) Representative western images and quantitative data of ROS production and apoptosis in 4 experimental groups (*n* = 5). (C, D) Representative Western blot images and quantitative data for the levels of BCL‐2, PAR‐2, and cytochrome *c* in four groups (*n* = 4). Data are presented as mean ± SEM and were analyzed using one‐way ANOVA and Tukey's post hoc test for panels B and C. Scale bars: 100 μm.

To examine the effect of Dex on stress serum–induced ROS production and apoptosis in HUVECs, we pretreated the cells with Dex or a combination with yohimbine for 30 min. The cells were then cultured in EBM‐2 containing 5% NS‐serum or S‐serum for 24 h, followed by assessments of ROS production and apoptosis. As shown in Figure 8A,B, the ROS and TUNEL staining results demonstrated that Dex reduced S‐serum–induced oxidative stress and apoptosis; these effects were diminished by α2‐adrenergic receptor blockade. Anticipating these outcomes, Western blot analysis of the lysates confirmed that Dex also rectified the harmful alterations in the protein levels of Bcl‐2, PAR‐2, and cytochrome *c* in HUVECs in response to S‐serum (Figure 8C,D).

## 4 Discussion

Through database analysis, we identified dexmedetomidine's effect on coagulation and subsequently investigated its impact on FeCl_3_‐induced chronic stress–related carotid thrombosis in mice. Experimental results demonstrated that Dex reduced stress‐related arterial thrombus formation induced by FeCl_3_ through the inactivation of the PAR‐2 signaling pathway. Based on findings from both in vivo and in vitro studies, we reached the following significant conclusions while decreasing: (1) Under experimental conditions, chronic stress increased plasma vWF and PAI‐1 levels and decreased plasma ADAMTS13 levels; however, Dex loading reversed this hypercoagulable state. (2) Dex corrected the imbalance of oxidative (gp 91^phox^) and antioxidant (SOD‐2) pathways, as well as apoptosis (C‐casp‐3 and cytochrome *c*) and anti‐apoptosis (Bcl‐2), along with intracellular signaling alterations (PAR‐2 and p‐Akt) caused by chronic stress. This correction reduced excessive inflammation (VCAM‐1, MCP‐1, ICAM‐1, TLR4, and TNF‐α), proteolysis (MMP‐2), and apoptosis, thereby protecting carotid artery endothelial integrity and minimizing the exposure of the vascular matrix, which in turn alleviated thrombus formation. (3) Blockade of AdR‐α_2_ with yohimbine diminished the vasculoprotective actions of Dex. (4) In vitro, Dex reduced the harmful changes in Bcl‐2, cytochrome *c*, and C‐casp‐3 levels caused by stress serum, decreasing ROS production and apoptosis in HUVECs. Thus, these findings provide evidence and a potential mechanistic explanation for the Dex‐mediated amelioration of arterial thrombosis, which is associated with reductions in oxidative stress, inflammation, apoptosis, and proteolysis, mediated by PAR‐2 signaling inactivation in mice exposed to our experimental stress conditions.

Endothelial cells are essential for maintaining vascular homeostasis, and their injury or apoptosis—leading to the exposure of the vascular basement membrane—is a key step in thrombosis in both humans and animals [28]. Oxidative stress is a major driver of cardiovascular cell apoptosis in various pathological conditions [29]. Dex has demonstrated anti‐apoptotic properties in a lung injury model [30]. Our immunofluorescence and immunohistochemistry observations revealed an increased number of endothelial cells in thrombotic lesions following Dex treatment. A recent review of fundamental studies emphasizes that Bcl‐2 suppresses caspase 3 activation and prevents the mitochondrial release of cytochrome *c*, both of which are associated with apoptosis [28]. A recent study noted that activation of PAR‐2 promotes renal injury induced by a high‐fat diet by inducing oxidative stress [31]. Our results show that AdR‐α2 agonist Dex treatment produced benefits on chronic stress‐induced harmful changes, including p‐Akt and PAR‐2, as well as apoptosis‐related proteins (Bcl‐2, cytochrome *c*, C‐casp3) and oxidative stress–related proteins (gp 91^phox^ and SOD‐2). These effects were diminished by AdR‐α2 antagonist yohimbine intervention, suggesting that the vasculoprotective actions of Dex are mediated, at least in part, through the negative regulation of the PAR signaling pathway. This concept is further supported by cellular experiments showing that Dex increased levels of Bcl‐2 protein while lowering levels of cytochrome *c* and PAR‐2 as well as reducing ROS production and HUVEC apoptosis; these effects were also diminished by Yoh.

Increasing evidence suggests extensive crosstalk between inflammation and the coagulation system [32]. After tissue injury, endothelial cells become activated and lose their anti‐thrombotic properties, while inflammatory mediators create a pro‐thrombotic environment [33]. Recent studies have shown that inhibiting the inflammatory response can reduce thrombosis [34]. PAR‐2 signaling promotes inflammatory responses in various cells and contributes to the development of inflammatory diseases [35]. Additionally, it was reported that PAR‐2 upregulates TNF‐α expression in endothelial cells and mediates vascular inflammatory damage through the TLR4/TNF‐α pathway [36]. On the other hand, TLR4 has been shown to enhance NF‐κB‐driven pro‐inflammatory gene expression through the EPCR‐dependent PAR2 activation in macrophages [37]. Recent study has demonstrated that PAR2 deletion attenuated NOD‐like receptor protein 3 (NLRP3) signaling, thereby mitigating cytokine storm [38]. In this study, stress upregulated PAR‐2 protein levels and increased the expression of inflammatory cytokines and chemokines (TNF‐α, ICAM‐1, VCAM‐1, TLR4, MCP‐1) as well as the protease MMP‐2. Interestingly, Dex rectified these harmful alterations, which were diminished by Yoh. Taken together, these data provide evidence that Dex‐mediated inactivation of PAR‐2 has an inhibitory effect on inflammation, contributing to the anti‐thrombosis induced in FeCl_3_‐induced mice under previous stress conditions.

A previous clinical study found that an imbalance between ADAMTS13 and vWF leads to a hypercoagulable state, promoting thrombosis in post‐COVID‐19 syndrome with stress [39]. Endothelial cells are the primary source of vWF production and secretion, as well as the main source of ADAMTS13, aside from the liver [40]. It has been reported that inflammation can induce thrombosis by disrupting the balance of vWF and ADAMTS13 through its effects on endothelial cells [41]. Activation of PAR‐2 has been shown to promote inflammation and renal injury in mice fed a high‐fat diet [31]. Our experiments observed an increase in plasma vWF and a decrease in ADAMTS13 levels in stressed mice, which were reversed following Dex treatment. Thus, AdR‐α2 activation by Dex exerts salutary effects on vascular endothelial damage and thrombus formation by reducing PAR‐2–mediated inflammation, thereby improving the imbalance of vWF and ADAMTS13 in mice under our experimental stress conditions.

PAI‐1 plays a crucial regulatory role in stress‐related metabolic diseases in humans and animals [22, 24, 42]. Our previous study showed that increased PAI‐1 expression contributes to insulin resistance and a pro‐thrombotic state [43]. TNFα, with and without ROS, has been shown to upregulate PAI‐1 expression in endothelial cells [44, 45]. We have demonstrated that Dex administration lowered the levels of TNFα, gp91^phox^, and SOD‐2 RNA and/or proteins in injured or stressed carotid arteries. Endothelial cell damage leads to the exposure of tissue factor, which triggers the activation of the coagulation cascade and thrombosis, while spontaneous fibrinolysis mediates fibrin degradation, thus limiting thrombus formation [46]. Collectively, Dex appears to suppress stress‐related arterial thrombus formation by reducing PAI‐1 activity through the reduction of TNF‐α‐ and oxidative stress production in mice under our experimental conditions.

In summary, our findings elucidate how chronic stress interferes with carotid arterial endothelial cell damage and demonstrate an inactivation of PAR‐2 signaling in the processes of stress‐related artery thrombus formation we report. Here, we have reported that Dex can ameliorate carotid arterial oxidative stress, inflammation, proteolysis, and endothelial cell apoptosis by activating PAR‐2 signaling, leading to a reduction in thrombus formation induced by FeCl_3_ in mice under our experimental conditions. Thus, Dex could represent a novel therapeutic approach to prevent arterial thrombosis in patients experiencing chronic psychological stress.

## Author Contributions

**Huazhen Wang:** conceptualization, investigation, formal analysis, methodology, writing – original draft. **Meiping Zhang:** investigation, methodology. **Minglong Xin:** data curation, methodology. **Xueling Yue:** data curation, methodology. **Jinshun Piao:** methodology. **Longguo Zhao:** methodology. **Hehui Bi:** validation. **Shiyan Wang:** validation. **Chunzi Jin:** validation, writing – review and editing. **Yongshan Nan:** validation, writing – review and editing. **Xianglan Jin:** methodology, supervision, writing – review and editing. **Xian Wu Cheng:** project administration, conceptualization, funding acquisition, supervision, writing – review and editing. All authors read and approved the article.

## Disclosure

The authors have nothing to report.

## Ethics Statement

All animal study protocols (Protocol No. YD20211120005) were approved by the Institutional Animal Care and Use Committee of Yanbian University and conducted in accordance with the Guide for the Care and Use of Laboratory Animals published by the U.S. National Institutes of Health.

## Conflicts of Interest

The authors declare no conflicts of interest.

## Data Availability

The data supporting the findings of this work are available in the Methods section.
